# The Spatiotemporal Evolutionary Trend and Driving Factors of the Coupling Coordinated Development between Regional Green Finance and Ecological Environment

**DOI:** 10.3390/ijerph19106211

**Published:** 2022-05-20

**Authors:** Tao Shi

**Affiliations:** Economics Institute, Henan Academy of Social Science, Fengchan Road 21, Zhengzhou 450002, China; taoshi_ccp@163.com

**Keywords:** green finance, ecological environment, coupling coordination rate, spatiotemporal evolution, driving factor

## Abstract

Based on the 30 inland provincial samples of China from 2003 to 2019, this article analyzes the evolutionary characteristics of the coupling coordination between green finance and the ecological environment (CCFE) using ArcGIS 10.5 software and employs the spatial Durbin model to analyze the driving factors of the CCFE. The results can be concluded as follows: (1) the CCFE of China is at a moderately low level, with a fluctuating upward trend. Spatially, it presents a spatial distribution pattern—higher in the east and lower in other regions. In terms of types, the regions of the CCFE are more in primary coordination and basic un-coordination and less in moderate un-coordination and moderate coordination. There are more regions of the green finance lagged type, and relatively few regions have achieved the financial ecological synchronization type. (2) The CCFE hotspots are concentrated in the Pearl River Delta, with a spatial “increase–decrease” development trend. Additionally, the CCFE cold spots are concentrated in the upper Yellow River Basin, with a relatively stable spatial scope. (3) The CCFE shows a positive spillover effect and accumulative delivery effect in the economic geospatial space. The population urbanization rate and the number of granted patent applications have a significant positive impact on the CCFE, and the percentage of secondary industries to GDP has a negative impact accordingly. Spatially, the percentage of secondary industries to GDP and the number of granted patent applications of nearby provinces in the economic geospatial space have a negative impact on the local CCFE. (4) The impact and spatial effect of different factors on the CCFE are obviously different. Finally, policy implications on the coordinated development of green finance and the ecological environment are also made.

## 1. Introduction

The ecological environment is the foundation of human survival and development; promoting the high-quality development of the ecological environment has received great attention. In the past 10 years, China has been committed to ecological environment construction and protection and has achieved remarkable results. According to the ecological environment bulletin in China (2020) and the annual report of the electricity industry in China (2021), in the “13th-Five-Year-Plan” period, China’s coal-fired power generation units meted the ultra-low emission limit and reached a total of 950 million kilowatts, the proportion of good days in the prefecture-level cities and above increased to 87.0% (target 84.5%), the average concentration ratio of PM2.5 in the prefecture-level cities that have not reached the standard decreased to 28.8% compared to that in 2015 (target 18%), the proportion of surface water with excellent quality increased to 83.74% (target 70%), and the safe utilization rate of polluted land reached 90%. Nowadays, green finance development in China has reached the international first-class level, and the effective development of the ecological environment cannot be separated from China’s earlier implementation of green finance policy [1]. Since China launched green loan products in 2012, China has gradually established an integrated green finance system, covering green credit, green bonds, green insurance, green security, and so forth. According to the annual report on green finance development in China (2021), up to June 2021, the accumulative issuance of green bonds in domestic China and abroad exceeded RMB 1.73 trillion, and the transaction value of the carbon emission rights trading market reached RMB 100 billion; this has strengthened the promotion of ecological environment protection and construction. However, there is a spatial unbalance in the development of green finance and the ecological environment in China [2]. China’s financial resources are mainly concentrated in the economically developed eastern regions, the carbon emission rights trading pilot regions. Except for Chongqing city and Hubei province, which are located in the central and western regions, Beijing, Shanghai, Guangdong, and Shenzhen are located in the eastern regions. Meanwhile, China’s ecological fragile regions are mainly located in the central and western regions, such as the northwestern arid region and the upper Yellow River Basin. In 2020, China announced that it would promote “3060” targets in the future and that green finance and ecological development would play important roles in the “3060” development process. Therefore, it is obviously significant to explore the coupling coordination development between green finance and the ecological environment (CCFE) and the driving forces.

The issues about green finance and the ecological environment have also attracted scholars’ attention. Based on 30 inland city samples in China, adopting the GMM model, H. Zhou & Xu (2022) [3] concluded that green finance has a negative impact on ecological development in China; there is an invert U-shaped relationship between green finance and the regions’ ecological development, and the inverted U-shaped relationship is more obvious in the central and western regions. Furthermore, R. Wang et al. (2022) [4] confirmed the conclusions above and considered that there is a nonlinear U-shaped relationship between green finance and regional ecological efficiency, especially in the central and western regions. In addition, some scholars have studied green finance and high-quality development [5] and green finance and economic growth [6]. The scholars have discussed the causal relationship between green finance and the ecological environment but have not explored the dependence and degree of dependence between the green finance system and the ecological environmental system. Based on the views above, this article will focus on the coupling coordinated relationship between green finance and the ecological environment and analyze the overall characteristics, spatiotemporal distributed patterns, spatial aggregation evolution characteristics, and driving forces of the CCFE. Additionally, the potential contributions are as follows: (1) Adopting the coupling coordination model, this article calculates the CCFE index of 30 inland sample provinces in China from 2003 to 2020 and, using ArcGIS 10.5, analyzes the spatiotemporal evolution of the overall characteristics and regional pattern characteristics of CCFE, showing the systematic development of CCFE in China. (2) Appling the spatial hot–cold model, this article analyzes the spatiotemporal evolution of different regions with a high or low value of CCFE. (3) Using the spatial Durbin model with a two-order lag period, this article analyzes the driving force affecting the CCFE and explores the spatial heterogeneity and spatial spillover effects of different factors. 

The rest part of this article is structured as follows. Section 1.1 provides a brief literature review on green finance and the ecological environment. Section 2 presents the calculated index of green finance and the ecological environment, the coupling coordination model, and the spatial Durbin model and describes the panel data used in this article. Section 3 reports the empirical estimation results, and we discuss the results in Section 4. Section 5 concludes the results and provides policy implications.

### 1.1. Literature Review

#### 1.1.1. The Appraisal of the Ecological Environment

Recently, scholars have had a consensus on the definition of ecological environment, and ecological environment appraisals have become a research hotspot. From the view of an evaluation system, most scholars have evaluated the ecological environment from the perspectives of comprehensive index evaluation and efficiency evaluation [7], and most related factors can be divided into the two aspects of natural factors and human factors. Among them, the natural factors include the forest cover rate, atmosphere variation, and nature protection region, and the human factors include urbanization and land use. A typical comprehensive evaluation system is similar to systems created by Yibo et al. (2021) [8], who constructed an ecological environment quality index system that includes natural ecology and social indicators, and Xiao et al. (2021) [9], who constructed an ecological environment evaluation index system that includes 13 aspects, such as per capita green area, per capita water resources, and energy consumption per unit of GDP. Meanwhile, a typical ecological environment efficiency evaluation system, such as the one studied by Zhang et al. (2022) [10], analyzes China’s environmental development efficiency; the input index includes the employed population, capital investment, and water use and the output index includes GDP, CO_2_ emissions, SO_2_ emissions, and soot emissions. Zhang et al. believe that environmental development in China shows a spatial distribution that is high in the east and low in other regions. Therefore, a comprehensive index evaluation system can better show the development of the ecological environment from more detailed perspectives, and we adopt this in this article.

Accordingly, scholars have used the principal component analysis method, the entropy method, the fuzzy Delphi analytic hierarchy process, and multiple normalization methods combined with pixel-phenology to evaluate the ecological environment [11]. Additionally, the typical method, such as Chang et al.’s method (2019) [12], uses spatial principal component analysis to analyze the ecological index in the upper Hanjiang river regions. Shi et al. (2020) [13] adopted the entropy method to calculate the regional ecological environment index in the tropical and subtropical regions in China from 2003 to 2016, and Y. Liu et al. (2022) [14] applied the DEA-SBM model to analyze ecological security. In addition, some scholars have used artificial intelligence and complex numerical optimization to analyze the ecological environment management problem [15].

#### 1.1.2. The Definition and Appraisal of Green Finance

Focusing on the improvement of the ecological environment, scholars have earlier studied green finance. Salazar (1998) [16] believed that financial innovation must base on environmental protection to improve social progress, achieving a dynamic balance between environmental protection and economic development. This is the first conception of green finance, and it emphasizes that the development target of green finance is environmental protection and social progress. Along with the more complex ecological environment development, environmental risk has begun to be more obvious, and the conception of green finance brings more detail and focus to environmental risk. Labatt and White (2002) [17] believed that green finance is a kind of financial innovation required to prevent environmental risk in financial instruments and product innovation. In 2016, China integrated the green development concept into the G20 agenda and reached a global consensus on the development of green finance; the target of green finance has been raised to emphasize the human survival situation, strengthening the ecological balance through financial services and product innovation [18]. Recently, the most recognized concept of green finance is to provide financial services for environmental protection, energy savings, clean energy, and green transportation, to support environmental improvement, resource savings, and efficient use, and to combat climate change [19,20], and we follow this definition in this article.

Based on the concept of green finance, few scholars have analyzed the evaluation of green finance from a quantitative perspective. Scholars have calculated the development of green finance using green bonds, green securities, and green insurance as the replacement variables of green finance [21]. Additionally, a single index cannot efficiently reflect the development level of green finance; some scholars have tried to construct a comprehensive evaluation system, including bonds and securities. [22]. In addition, some scholars have appraised green finance development efficiency from the input and output perspectives [23].

#### 1.1.3. The Relationship between Green Finance and the Ecological Environment

Scholars believe that green finance has efficiently promoted ecological environment development [24]. In one aspect, the government guides the development direction of the market to clean technology, such as carbon capture, tidal energy, and nuclear energy [25]. Meanwhile, using environmental regulation tools such as an environmental tax, the government can dispose of environmentally destructive actions to promote the high-quality development of the ecological environment. In another aspect, sustainable energy investment can be promoted to advance industrial decarbonization [26] using green financial tools, such as green bonds [27]. Additionally, it can also guide the green low-carbon transformation of energy consumption structures and industry structures [28] to promote the green low-carbon development of the economy, reducing the environmental pollution caused by economic and social development. Especially in the COVID-19 pandemic period, green financial policies such as carbon pricing and tradable green certificates can efficiently reduce the financial cost and counteract the negative shock of the pandemic [29]. In addition, green finance is a key factor for high technology to drive green growth, keeping resource prices and economic development stable [30].

However, some scholars believe that green finance does not bring clean energy but that green financial investments are only impelled by the demand for clean energy [31], and environmental regulations will constrain the role of green finance [32]. Therefore, combined with the modern concept of green finance, we can see that green finance emphasizes the coordinated development of financial activities, environmental protection, and ecological balance to realize the continuous development of economies and society [33], and green finance and the ecological environment show a merged and interactive developmental relationship. Indeed, from 1998 to 2018, the ecological environment of only one-third of China has improved [34] with the rapid development of green finance development. Therefore, according to the study above, we propose hypotheses H1 and H2:

**Hypothesis 1** **(H1)**. *The mutual and interactive developmental relationship between green finance and the ecological environment in China is at a low level with spatial heterogeneity.*

**Hypothesis 2** **(H2)**. *There are some factors that constrict the mutual and interactive developmental relationship between green finance and the ecological environment in China.*

#### 1.1.4. Summary

**Figure 1:**
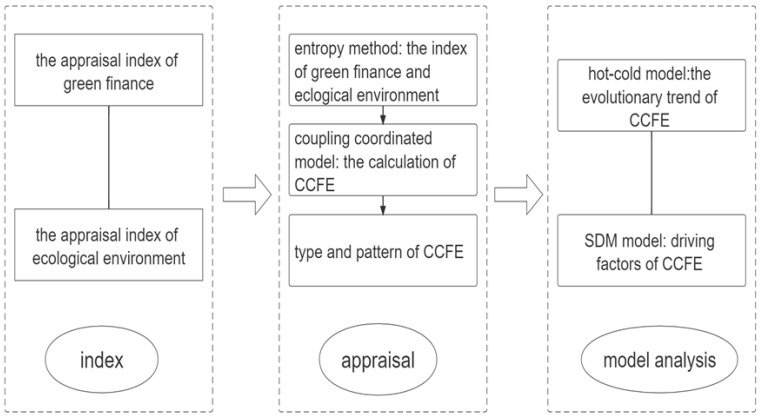
The technological path of this article.

As discussed above, scholars have analyzed the related issues on green finance and the ecological environment from different perspectives, with more study on the concept, impact, and appraisal and less on the relationship between green finance and the ecological environment. Few scholars have explored green finance and green development, economic growth, and ecological development efficiency, and we lacked a study on the CCFE. Therefore, based on the 30 inland provincial samples from 2003 to 2020 in China, this article will systematically analyze the overall characteristics, spatiotemporal evolution, and driving factors of the CCFE to promote green finance and the ecological environmental development of China in the “14th-Five-Year-Plan” period. Figure 1 shows the technological path of this article.

## 2. Materials and Methods

### 2.1. The Calculation of Green Finance and the Ecological Environment

Referring to the current research [35,36], adhering to the principle of systematicness, scientificity, comprehensiveness, and accessibility, combined with the reality of China’s green finance and ecological development, we constructed a comprehensive evaluation index system of green finance and the ecological environment. Table 1 shows the index and statistical descriptions; the values presented in Table 1 are for regions in China.

Green finance aims to support economic activities, such as environmental improvement and tackling climate change [37], by using comprehensive tools. Therefore, we chose green securities, green industries, green credit, and green investments to comprehensively calculate the development level of green finance, calculating the index of the market value proportion of environmental protection enterprises to total enterprises; the proportion of fixed asset investments in water conservancy, environment, and the public facilities management industry to total social fixed asset investment; the proportion of interest expenses in the output of six high-energy-consumption industries to the total industry; and the proportion of completed investments in industrial pollution to GDP correspondingly. Among them, according to the industrial category in the software of Tonghuasun, we chose the environmental protection pattern of the enterprise as air governance, water governance, solid waste governance, comprehensive environment governance, and environmental protection equipment.

The ecological environment is a complex ecosystem related to the sustainable development of society and the economy, and it is a comprehensive reflection of environmental pollution and environmental governance [38]. Therefore, we chose the pollution intensity of the ecological environment, the governance intensity of the ecological environment, the construction level of the ecological environment, and energy intensity to calculate ecological environmental development. Among them, the pollution intensity of the ecological environmental represents the ecological polluted level caused by different types of pollution, such as air and solid waste; an index of total industrial wastewater discharge, total industrial waste air emission discharge, total industrial solid waste production, and the number of environmental pollution incidents was chosen. Ecological environment governance intensity reflects the governance performance of the different roles of government, enterprises, and individuals [39,40]; an index of the percentage of fiscal expenditure on environmental pollution to GDP, the percentage of environmental pollution governance investment to GDP, the harmless treatment rate of domestic waste, total industrial wastewater treatment, the city sewage treatment rate, the treatment capacity of industrial waste gas treatment facilities, and the comprehensive utilization rate of industrial solid waste was chosen. The construction level of the ecological environment is the realistic manifestation of ecological environment condition improvements, such as regional forest cover, and an important foundation promoting ecological environment development [41]; an index of the area of natural protection, public green area per capita, green space rate of built-up area, and forest coverage was chosen. Traditional resources such as oil are an important source that leads to ecological environment pollution, while clean energy, such as electricity, can efficiently improve the ecological environment. Different production modes also play an important role in changes in the ecological environment [42]; an index of electricity consumption, energy consumption, energy consumption per unit GDP, total apparent CO_2_ emissions, and carbon intensity was chosen.

### 2.2. The Analysis of CCFE and Driving Force

#### 2.2.1. The Calculated Method of CCFE

Compared to comprehensive calculated methods, such as the analytic hierarchy process, the entropy method can avoid subjective factors impacting the results and more objectively evaluate the index [43,44]. Therefore, based on the entropy method, we calculate the ecological environmental development level *iecolg_i_* in year *i* as follows:

(1) $$iecolg_{i}=\sum_{j=1}^{m}w_{j}\times x_{i}^{\prime }$$

In Formula (1), *w_j_* represents the weight of different samples; $x_{i}^{\prime }$ refers to the standardized index and is calculated as follows:

(2) $$\left\{\begin{array}{l} x_{i}^{\prime }=\frac{x_{i}-\min\{x_{1},\ldots ,x_{n}\}}{\max\{x_{1},\ldots ,x_{n}\}-\min\{x_{1},\ldots ,x_{n}\}}\\ p_{i}=(1+x_{i}^{\prime })/\sum_{i=1}^{n}\left(1+x_{i}^{\prime }\right)\\ e_{j}=-k\sum_{i=1}^{n}p_{i}\times \ln(p_{i}),k=1/\ln(n)\\ w_{j}=d_{j}/\sum_{j}^{m}d_{j},d_{j}=1-e_{j} \end{array}\right.$$

In Formula (2), *x_i_* refers to the index value correspondently. A change in the numerator of $x_{i}^{\prime }$ to (max{*x_1_*, …, *x_n_*}-*x_i_*) will negatively standardize the value of *x_i_*. Additionally, p_i_ represents the weight of the sample index, *e_j_* refers to the information entropy in index *j*, *d_j_* is the utility of index j, and n is the sample number; lastly, we calculate the weight of different indices, *w_j_*. Similarly, we calculate the green finance development index *igreen_i_* using the same method above.

#### 2.2.2. The Calculation of Coupling Coordination

Referring to the definition of capacity coupling and the coefficient model in physics [45,46] and setting the couple value between green finance and the ecological environment, we obtained the coupling appraisal model between green finance and the ecological environment; the couple value *C_j_* in region *j* is calculated as follows:

(3) $$C_{j}={\left\{\frac{S_{ieco\mathrm{lg}}\times S_{igreen}}{{\left(\frac{S_{ieco\mathrm{lg}}+S_{igreen}}{2}\right)}^{2}}\right\}}^{k},\left(k\geq 2\right)$$

In Formula (3), *k* represents the number of coupling indices, and setting *k* = 2. However, the couple level cannot efficiently reflect the high and low levels of the coordinated development between green finance and the ecological environment, especially since it is difficult to represent the “overall efficacy” and “coordination effect” of regional green finance and the ecological environment [47]. Additionally, we need to calculate the CCFE of *iccdn_j_*, as follows:

(4) $$iccdn_{j}=\sqrt{c_{j}\times t_{j}},\;t_{j}=\alpha S_{iecolg}+\beta S_{igreen}$$

In Formula (4), *α* and *β* are the important indicators of green finance and the ecological environment, respectively. In the current global economic development condition, green finance is as important as the ecological environment, setting *α* = *β* = 0.5. Referring to the current research, we divided the CCFE types, as shown in Table 2.

#### 2.2.3. The Spatial Analysis Method

Firstly, using the hot–cold model, we analyze the spatiotemporal evolution of the CCFE. The hot-cold model can efficiently reflect the variation of variables with high or low values [48,49] and remedy the shortcoming of the global or local Moran I index. Therefore, we adopt the Getis-Ord Gi* index to depict the spatiotemporal evolutionary characteristics of the CCFE, as follows:

(5) $$Gi*=\frac{\sum_{i=1}^{n}\sum_{j=1}^{n}w_{ij}x_{i}x_{j}}{\sum_{i=1}^{n}\sum_{j\neq 1}^{n}x_{i}x_{j}}$$

In Formula (5), *x_i_* > 0, *x_j_* > 0, and *w_ij_* refers to the spatial weight matrix reflecting the relationship between spatial units, calculated by the particle distance between provincial capital cities as: *w_ij_* = 1/*d_ij_*. Meanwhile, it is the hot regions of the CCFE when Gi* > 1.96, and it is the cold regions of the CCFE when Gi* < 1.96. In addition, we observe the hot–cold characteristics of spatial unit *i* using the local Getis-Ord Gi* index, as follows:

(6) $$Gi*=\frac{\sum_{j\neq i}^{n}w_{ij}x_{j}}{\sum_{j\neq i}^{n}x_{j}}$$

Using the spatial hot–cold model, we can explore the evolutionary trend of the CCFE, but we do not know why the evolutionary trend of the CCFE in different regions is different, and we need to further analyze the driving forces of the CCFE. In fact, under the national strategy of strengthening and promoting the financial services supporting ecological environmental protection, the spatial correlation of the CCFE is more closed than before [50]. Additionally, the spatial Durbin model (SDM) can better reflect the impacted factor from spatial and temporal perspectives [51]. Therefore, we establish an SDM model using the eco-geographical distance matrix (*W*), as follows:

*ln(iccdn_it_) =α* + *βlnX_it_* + *ρW_ij_ln(iccdn_it_)* + *μ_i_* + *υ_i_* + *ε_it_* (7)

*ε_it_* = *λW_ij_ε_it_* + *η_it_* (8)

In Formulas (7) and (8), *μ_i_* refers to the regional effect, *υ_i_* refers to the time effect, *ε_it_* represents the random error item, and *ρ* is the spatially lagged item coefficient. *W_ij_* refers to the spatial weight matrix in line *i* and column *j*, and it is defined as the product of the economic weight matrix and the geographical weight matrix; the economic weight matrix is calculated by the reciprocal difference in absolute values of GDP per capita. *X* refers to the serial impacted factors; *η* refers to the random item. According to the current reference, we analyze the driving factor of the CCFE from the perspectives of economic development, industrial structure, population, and investment. Among them, the industrial structure is calculated by the proportion of secondary industries to GDP (rsecd). Compared to the other variables reflecting the technological innovation level, the number of granted patent applications can realistically reflect the regional technological innovation level [52]; hence, the number of granted patent applications was chosen to reflect the regional technological innovation level (rpatent). The high-education resource is the human resource of a region’s green finance and ecological environment development [53], and the average number of students in high school was chosen to reflect regional human resources (nedu). Investment is an important driving force promoting regional economic development, and it is an important support for improving regional ecological environment development [54]; the proportion of fixed investments to GDP (rfixed) was chosen to reflect the regional investment level. Population is a key variable in changing the ecological environment. The overloaded ecological pressure of population will restrict ecological environment development, while a higher population will also promote regional economic development [55]; the population urbanization rate (rurban) was chosen to reflect regional economic development and population concentration intensity.

### 2.3. The Sources of Materials

Without special statements, the data used in this article are from the China Statistics Yearbook, the China Financial Statistics Yearbook, the China Fiscal Statistics Yearbook, and the China Ecological Statistics Yearbook. Meanwhile, excluding the sample available in hard copy from Tibet, we constructed panel data with 30 inland samples from 2003 to 2020 in China. Additionally, for the vacancy value in the samples, the five-year moving average value was used to supplement the information. The logarithmic method in all variables was used to reduce the impact of variation, and Table 3 shows the statistic descriptions.

## 3. Results

### 3.1. The Spatiotemporal Evolution of the Regional CCFE

#### 3.1.1. The Spatiotemporal Development Trend of the Regional CCFE

**Figure 2:**
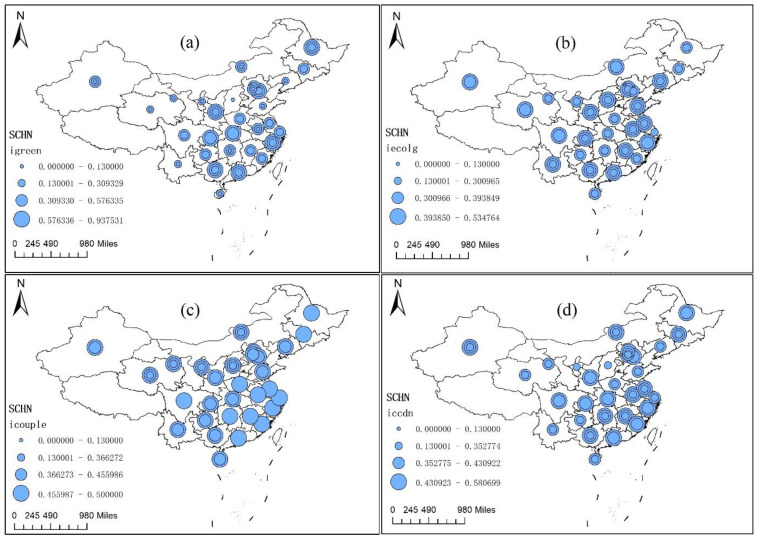
The evolutionary trend of related indices from 2003 to 2020. Note: (1) The symbol (**a**–**d**) refers to the green finance index, the ecological environment index, the coupling index between green finance and the ecological environment, and CCFE respectively.

Using the Jackknife nature breakpoint method, Figure 2 shows the spatiotemporal distribution of the green financial index, the ecological environmental development index, the coupling index, and the regional CCFE in China. Among them, the circle size represents the high or low level of the index, and the number of circles indicates the variation of the CCFE.

Figure 2a shows the spatiotemporal evolution of the green finance index. Generally, the average value of the green finance index ranges between 0.13 and 0.93, indicating that green finance development in China is at a middle and low level, with a fluctuating upward trend. Recently, China’s economy has entered a new stage of high-quality development, strengthening the financial support for the green low-carbon transformation of industry and life, and green finance development has improved steadily. Spatially, there are fewer regions with low green finance indices; these are mainly concentrated in the eastern economic development regions and the green development pilot zones in the central and western regions. The eastern region has higher economic development to promote the high-quality development of green finance, with more green financial institutions and stronger financial innovation ability. The green financial pilot zones, such as Hubei province and Chongqing city, had earlier explored the development of green finance and accumulated development experiences to promote green finance development. Notably, the development level of green finance in most regions has obviously changed, while Hubei province and Chongqing city are stably developed, with a high development level. In 2011, Hubei province became a trading pilot zone of carbon emission rights, and it has vigorously explored the trading model of carbon emission rights, the Clean Development Model, and promoted regional green finance development. Similar to Hubei province, Chongqing has also been a trading pilot zone of carbon emission rights in China since 2011, and, in 2017, Chongqing issued a “13th-Five-Year-Plan” to build a national functional financial center and was the first to take the lead in launching a pilot project of green finance development at the provincial level, especially protecting the ecological environment of the Yangtze River; accordingly, Chongqing’s green finance development level is higher than in other regions.

Figure 2b shows the spatiotemporal evolution of the ecological environment index. Generally, the average value of the ecological environment index ranges from 0.19 to 0.53, indicating that ecological environment development in China is also at a middle and low level, with a steady upward trend. Recently, China has vigorously promoted environmental protection and successively issued many environmental protection laws and regulations to strengthen the governance of air pollution and carbon emissions; China has also implemented the construction of shelter forest systems in key regions such as the Yangtze River and coastal areas, construction projects of natural forest protection, sandstorm governance in “Beijing-Tianjin-Hebei”, and fast-growing forests, which have obviously improved the ecological environment. Spatially, there are more regions with relatively higher ecological environment indices, and these regions are mainly concentrated in the southeastern and northeastern regions. Additionally, there are fewer regions with relatively lower ecological environment indices; these are mainly concentrated in the central regions. In recent years, China has strengthened the ecological environmental governance in the regions such as the Pearl River, enhanced the supervision of ecological environment protection, promoted the green production transformation of core carbon reduction, and improved the ecological development in related regions. At the same time, the central regions with more natural resources, such as Shanxi province and Henan province, have had more pressure on their ecological environments, such as air pollution and wastewater governance, which have restricted the development of their ecological environments.

Figure 2c shows the spatiotemporal evolution of the coupling index between green finance and the ecological environment. Generally, the average value of the coupling index between green finance and the ecological environment ranges from 0.13 to 0.51, indicating that the coupling index between green finance and the ecological environment in China is at a middle and low level, with a steady upward trend. Recently, in order to improve the ecological environment, China has successively issued many policies, such as green bond guidelines, and continuously constructed the green finance system, including green credits, green stocks, green development funds, and green insurance. Additionally, the objective reality faced by the ecological environment also provided a basis for green finance innovation, deepening the mutual relationship between green finance and the ecological environment. Spatially, the number of regions with higher coupling indices is large, with small changes, and they are mainly located in the eastern developed regions, owing to the developed finance development level and low-carbon industrial structure. The number of regions with lower coupling indices is small, with obvious changes, and they are mainly located in the northwestern regions and depend on less-green finance resources.

Figure 2d shows the spatiotemporal evolution of the CCFE. Generally, the average value of the CCFE ranges from 0.10 to 0.60, indicating that the CCFE level in China is at a middle and low level and in a primary coordinated stage, with a steady upward trend; Hypothesis 1 is verified. Additionally, the results indicate that the interactive effect between green finance and ecological development is more obvious. In fact, depending on the development of the ecological environment, the innovation development of green finance in China has been at the forefront of the world. Up to 2020, green loans in China reached RMB 12 trillion, with a proportion of 6.9% of total loans, and green bond stocks also reached RMB 813.2 billion, with zero default cases. Additionally, due to green finance innovations, such as the carbon emission rights quota trading rule (CEA) and the securitization of environmental rights and interests, the ecological environment has obviously improved, especially in carbon reduction. As of 2020, the trade of CEA reached 76.92 million tons. Moreover, the production methods of China are more obviously green. In 2021, the new grid-connected capacity of photovoltaic power generation in China reached 54.88 million kilowatts, and the proportion of clean energy in total energy consumption reached 25.5%, increasing by 1.2% compared to 2020. Among them, the percentage of coal consumption declined to 56.0%, decreasing by 0.95% compared to 2020. Spatially, the number of regions with higher CCFE indices is small, and the regions with stable CCFE indices are mainly located in the eastern developed regions and western green finance pilot zones, owing to the related high development of financial and low-carbon industrial structures. The regions with more fluctuating CCFE indices are mainly located in the central regions, restricted by the high-carbon industry, similar to the above. The number of regions with lower CCFE indices is large, with obvious changes, and they are mainly located in the central and western regions. Similar to the above, the green finance resources in the central and western regions cannot efficiently match the industrial green transformation development as they are limited by the higher proportion of high-carbon industries and relatively fewer financial resources.

#### 3.1.2. The Spatiotemporal Evolution of Regional CCFE Type

**Figure 3:**
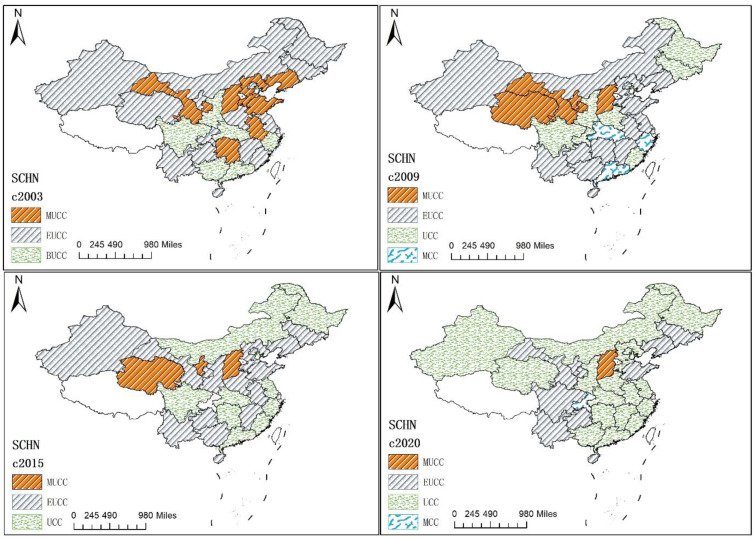
The evolutionary trend of CCFE type in years 2003, 2009, 2015, and 2020. Note: (1) The abbreviations of MUCC, EUCC, UCC, and MCC are the CCFE types mentioned above.

Figure 3 shows the evolutionary trend of CCFE types in the years 2003, 2009, 2015, and 2020, respectively. Generally, the CCFE level has steadily increased, showing a relatively concentrated distribution. In 2003, there were fewer regions with the primary type of CCFE, such as Zhejiang and Sichuan, with a relatively spatially concentrated distribution, and more regions with the basic un-coordination type of CCFE, such as Qinghai and Yunnan. There is also a middle un-coordination type of CCFE, such as Gansu and Shanxi, with a spatially concentrated contiguous distribution. In 2009, the regions with the middle un-coordination type and the primary coordination type continuously narrowed, while the regions with the basic un-coordination type and the middle coordination type gradually expanded; accordingly, the spatial distribution is similar to 2005. Among them, the number of regions with the basic un-coordination type expanded the most, and the number of regions with the middle un-coordination type narrowed the least. In 2015, the regions with primary coordination continuously expanded, while the regions with the middle coordination type and the basic un-coordination type obviously narrowed. Among them, the number of regions with the primary coordination type expanded the most, showing a more spatially concentrated contiguous distribution. In 2020, the number of regions, such as Chongqing, with the middle coordination type and Guangxi and Jiangxi with the primary coordination type, expanded to 1 and 18, respectively, and the number of regions, such as Sichuan and Liaoning, with the basic un-coordination type and Shanxi with the middle un-coordination type narrowed to 10 and 1 respectively. Among them, the number of regions with the primary coordination type expanded the most, and the number of regions with the middle un-coordination type narrowed the least. Spatially, the regions with the middle coordination type are scattered and located in the western developed provinces. The regions with the primary coordination type are contiguously concentrated and distributed in the eastern coastal provinces. The regions with the basic un-coordination type are concentrated and distributed in the southwestern and northeastern provinces, and the regions with the middle un-coordination type are scattered and located in the central provinces.

Further, according to Table 3, Table 4 shows the CCFE pattern in 2003, 2009, 2015, and 2020. In Table 4, the spatial distributions of the four different CCFE patterns are quite different in the samples. The ecological lagged regions are located in the eastern economically developed areas, the green finance development lagged regions are located in the central and western areas, and the green finance and ecological environment synchronized regions are mainly located in the western areas. Additionally, the green financial lagged pattern is the main pattern of the CCFE; the number of regions in the green finance lagged pattern, the ecological environment lagged pattern, and the green finance and ecological environment synchronized pattern are 15 (more than), 14 (less than), and 8 (less than), respectively. Meanwhile, regions with the ecological environmental development lagged pattern and the green finance and ecological environment synchronized pattern are mainly with the primary coordination type, and regions with the green finance lagged pattern are mainly in the basic un-coordination pattern. Therefore, the green finance lagged pattern is the main pattern of the CCFE in the samples and less in the synchronized pattern, indicating that there are more regions in the un-coordination relationship between green finance and the ecological environment.

#### 3.1.3. The Spatial Agglomerated Evolutionary Trend of the CCFE

**Figure 4:**
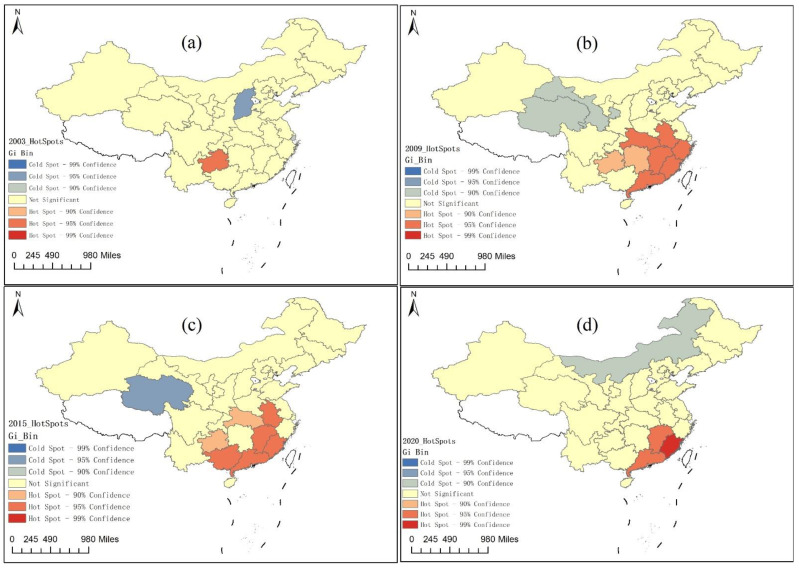
The spatial agglomerated evolutionary trend of hot–cold spot regions of the CCFE in the years 2003, 2009, 2015, and 2020, respectively. Note: (1) The symbol (**a**–**d**) refers to the green finance index, the ecological environment index, the coupling index between green finance and the ecological environment, and CCFE re-spectively.

Furthermore, Figure 4 shows the spatial agglomerated evolutionary trend of hot–cold spot regions of the CCFE in 2003, 2009, 2015, and 2020. In 2003, Guizhou province in the western region was in the high-value cluster of the CCFE, forming a hotspot region for the coordinated development of green finance and the ecological environment. Guangxi province in the western region was in the low-value cluster of the CCFE, forming a cold spot region for the coordinated development of green finance and the ecological environment. In 2009, the spatial distribution of the hot–cold spot regions of the CCFE changed in an obvious manner. Zhejiang province, Fujian province, and Guangdong province in the southeastern economically developed regions; Hubei province, Anhui province, and Jiangxi province in the central region; and Guizhou province in the western region were in the high-value cluster of the CCFE, forming a hotspot region for the coordinated development of green finance and the ecological environment. Gansu province and Qinghai province in the western region were in the low-value cluster of CCFE, forming a cold spot region for the coordinated development of green finance and the ecological environment. In 2015, the spatial distribution of the hot–cold spot regions of the CCFE also changed in an obvious manner. Fujian province and Guangdong province in the southeastern economically developed regions; Anhui province, Hubei province, and Jiangxi province in the central region; and Guangxi province and Guizhou province in the western region were in the high-value cluster of the CCFE, forming a hotspot region for the coordinated development of green finance and the ecological environment, with no cold spot regions. In 2020, the spatial distribution of the hot–cold spot regions of the CCFE changed greatly. Fujian province and Guangdong province in the southeastern economically developed regions and Jiangxi province in the central region were in the high-value cluster of the CCFE, forming a hotspot region for the coordinated development of green finance and the ecological environment. The Inner Mongolia Autonomous Region in the western region was in the low-value cluster of the CCFE, forming a cold spot region for the coordinated development of green finance and the ecological environment.

Therefore, the hot spot regions of the CCFE are mainly located in the eastern region, while cold spots are mainly distributed in the western region; the results are basically consistent with the spatial distribution of the three patterns of the CCFE in Figure 2. Compared to other regions, the eastern coastal region is an area with a high economic development level, especially with more green financial resources and lower carbon industrial structures; most cities there are hot spots of the CCFE.

### 3.2. The Driving Force Analysis of CCFE

#### 3.2.1. The Analysis of the Benchmark Model

According to the result of the Hausman test, reducing the impact of the endogenous problem, this study controlled the time effect and spatial effect of the samples and adopted the fixed-effect SDM model with a two-order lag and eco-geographical matrix to estimate the results of the benchmark model. Meanwhile, in order to test the stability of the estimation results, the stepwise regression model was used; Table 5 shows the estimation results of the benchmark model. In Table 5, the spatial correlation coefficients of spatial rho are significantly positive at the 1% confidence level, indicating that the coordinated development of green finance and the ecological environment in the eco-geographical adjacent province is conducive to promoting the coordinated development of green finance and the ecological environment at the local level, showing a significantly positive spatial spillover effect.

The lag term of the CCFE is significantly positive at the 1% confidence level, indicating that the CCFE in previous periods had a significant positive impact on the development of the period and the coordinated development of green finance and the ecological environment has a positive cumulative transfer effect. As mentioned above, the CCFE in China showed a steady upward trend and objectively confirmed the positive cumulative transfer effect. The coordinated development model of green finance and the ecological environment in previous periods can provide an improvement and innovation basis in the future by processing the “improving–optimizing–improving-…” process to continuously promote the coordinated development of green finance and the ecological environment.

The population urbanization rate is significantly positive at the 1% confidence level, indicating that the increase in urbanization rate is conducive to promoting the CCFE. Currently, China is continuously developing new urbanization without sacrificing the development of agriculture, ecology, and the environment and promoting infrastructure integration and public services equalization between urban and rural spaces. Additionally, ecological environment development will enter into a healthy track and improve accordingly when the increase in the population urbanization rate matches the corresponding social resources, such as the economy, medical care, education, and material resources such as water and energy. Therefore, the development of new human-centered urbanization will improve the development of the ecological environment and be beneficial to promoting the coordinated development of green finance and the ecological environment.

The percentage of secondary industries to GDP is significantly negative at the 1% confidence level, indicating that the increase in the proportion of secondary industries is not conducive to the development of the CCFE. The proportion increase of secondary industries will continuously enhance ecological pollution pressures such as carbon emissions if it ignores the structure optimization of raising the percentage of newly strategized industries, such as the manufacturing service industry, and green low-carbon transformation, such as the production of clean energy. Additionally, the customized secondary industries with high carbon emissions and high waste energy will increase the compliance risk and operation risk of market actors such as enterprises, reducing the green credit motion of financial institutions such as banks, and this will not favor the coordinated development of green finance and ecological development. Meanwhile, the spatially lagged coefficients W×lnrsecd are significantly negative at the 10% (at least) confidence level, indicating that the proportion increase of secondary industries in eco-geographical adjacent provinces will siphon local provincial resources such as green finance, ecological talents, and technology and be detrimental to the local development of the CCFE.

The number of granted patent applications is significantly positive at the 10% confidence level, indicating that the development of the regions’ technological innovation will promote the development of the CCFE. Improving regional technological innovation, on one hand, can enhance the development quality of the ecological environment, using clean energy and green buildings such as tidal energy, nuclear energy, carbon capture, clean buildings, and incineration flue gas purification technology. On the other hand, embedding advanced technology such as blockchain, cloud computing, artificial intelligence, and big data in the innovation of financial products and services can efficiently improve the efficiency of green finance by servicing ecological environment development and realizing the efficient and coordinated development of green finance and the ecological environment. The spatially lagged coefficients W×lnrpatent are significantly negative at the 5% confidence level, indicating that the improvement of technological innovation in eco-geographical adjacent provinces will siphon local, provincial resources such as talents and technology and be detrimental to the local development of the CCFE. Therefore, Hypothesis 2 is verified.

#### 3.2.2. The Heterogeneity Analysis

Furthermore, we analyzed the heterogeneity of driving forces on the CCFE. Time-wise, we set the year 2014 as the time split point, considering the environment protection law of China was formally implemented in 2015, and explored the impact difference of this driving force before or after the implementation of the law. Spatially, we divided the samples into four regions—east, center, west, and northeast—according to the National Development and Reform Commission of China and analyzed the impact of the heterogeneity of different factors on different regions. Table 6 shows the estimation results.

From the perspective of time heterogeneity, in the years after 2015, the percentage of secondary industries and the percentage of fixed investments are significantly negative at the 1% confidence level, indicating that the increase in the proportions of secondary industries and fixed investments is not conducive to the development of the CCFE after 2015. After 2015, the implementation of the national environment protection law obviously increased the trade cost of illegal environmental activities such as environmental disruption and ecological pollution. Currently, the industry proportion of high energy consumption, high pollution, and wastewater in secondary industries is also high in China; increasing traditional secondary industries will raise the operational cost of enterprises and enhance the risk pressure of green financial institutions such as commercial banks, decoupling the development of the CCFE. In addition, the spatially lagged coefficients W×lnrsecd are still significantly negative at the 1% confidence level and are not affected by the time changes.

From the perspective of regional heterogeneity, the population urbanization rate is significantly positive at the 10% confidence level in the eastern region and the northeastern region and significantly negative in the western region. The result indicates that the increase in the population urbanization rate in the new urbanization is conducive to the development of the CCFE in the eastern and northeastern regions and not in the western region. As mentioned above, the eastern region in China is an economically developed area, and the development of new urbanization can promote the high-quality development of the CCFE, while the northeastern region has incremental development at a low level. Meanwhile, the western and central regions face serious constraints of ecological protection issues, especially the low-carbon transformation pressure of green industries, ecological fragility such as soil erosion, and an overly high proportion of heavy industries in the upper Yellow River Basin; the slowing construction of new urbanization has restricted the development of CCFE correspondingly. Additionally, the spatially lagged coefficients W×lnrurban in the central and western regions are still significantly negative at the 10% confidence level and significantly negative at the 10% confidence level, indicating that the increase in the population urbanization rate in eco-geographically adjacent provinces has a spatial positive spillover effect on local provinces in the central and western regions and a negative spatial spillover effect on the northeastern region.

The number of granted patent applications is significantly positive at the 1% confidence level in the western region, indicating that the development of technological innovation is conducive to the development of the CCFE in the western region. Additionally, the spatially lagged coefficients W×lnrpatent in the western region is significantly negative at the 5% confidence level, indicating that the development of technological innovation in eco-geographically adjacent provinces has a spatial negative spillover effect on the local provinces; the siphon effect is more obvious in the western region.

The number of students of high education per 100,000 inhabitants in the population in the eastern region is significantly negative at the 1% confidence level and significantly positive at the 5% confidence level in the central and western regions, indicating that there should be more emphasis on the structure and not the scale of talents in the eastern region; strengthening the scale of talents in the central and western regions is conducive to promote the development of the CCFE correspondingly. Additionally, the spatially lagged coefficients W×lnnedu in the eastern region are significantly negative at the 1% confidence level, indicating that the expansion of high education resources in the eastern region has a spatial positive spillover effect, promoting the development of the CCFE in local provinces. Similar to the results above, the proportion of fixed investments is significantly negative at the 10% confidence level in the eastern region, western region, and northeast region, and the spatially lagged coefficients W×lnrfixed are significantly negative at less than the 10% confidence level.

#### 3.2.3. The Analysis of the Spatial Spillover Effect

In addition, according to Formula (7) and the results in Table 5, Table 7 shows the total spatial effect, direct effect, and indirect effect of the CCFE.

The direct effects of the population urbanization rate in the short and long term are significantly positive at the 1% confidence level, indicating that the increase in the population urbanization rate can promote the development of the CCFE in the inner regions. The total effect, direct effect, and indirect effect of the proportion of secondary industries in the long and short term are significantly negative at less than the 10% confidence level, indicating that the enhancement of the proportion of secondary industries is not conducive to CCFE development in the overall, local, and eco-geographically adjacent regions. Accordingly, the direct effect of the proportion of secondary industries is larger than the indirect effect, showing that the restricted effect is obvious in external regions. The direct effect of the number of granted patent applications in the short term is significantly positive at the 5% confidence level, and the total effect and indirect effect in the short term are significantly negative at the 5% confidence level, indicating that the improvement of technological innovation is not conducive to the development of the CCFE in the short term. Particularly, the inhibitory effect of technological innovation is especially obvious in the local region, while the promoted effect is significant in the eco-geographically adjacent region. The total effect and indirect effect of the proportion of fixed investments in the short term are significantly negative at the 5% confidence level, indicating that increasing the proportion of fixed investments will restrict the development of the CCFE in the overall sample and eco-geographically adjacent provinces.

## 4. Discussion

This article analyzes the evolutionary trend of the CCFE from 2003 to 2020 in China, and the results show that the CCFE in China is at a primary stage, with a fluctuating upward trend. Spatially, the CCFE shows spatiotemporal evolutionary characteristics, such as higher values with fewer changes in the eastern region and lower values with more changes in other regions. Most regions are dominated by the primary coordination type, followed by the basic un-coordination type, and there are fewer regions with the middle un-coordination type and the middle coordination type. Correspondingly, the green finance lagged pattern is the main type in all samples, and few regions have reached the economic-ecological synchronized pattern. Consistent with Zhang et al. (2022) [56], it is believed that green finance and environmental performance in China are in the primary coordinated stage, with a spatial distribution pattern of orderly decrease from east to west. Using the Getis Ord Gi* index, we analyzed the hot–cold spot evolutionary trend of the CCFE, and the result showed that the hot spots of CCFE are concentrated in the eastern region, such as the Pearl River, with an “increase–decrease” development trend in the spatial space, while the cold spots are located in the western region, such as the upper Yellow River Basin, with a stable spatial space. Similar to the results of Lv et al. (2021) [57], it is believed that there is a development gap in green finance, especially in the eastern region, with a polarization phenomenon.

The spatial Durbin model was used to analyze the driving force of the CCFE. The development of the CCFE in eco-geographically adjacent provinces shows a significantly positive spatial spillover effect and a cumulative transfer effect. Enhancing the population urbanization rate is conducive to the development of the CCFE, and the promoted effect is obviously significant in the eastern and northeastern regions, while it is negative in the western region. Actually, the development of the ecological environment and urbanization in the western regions is relatively lower than in other regions [58]; this has restricted CCFE development accordingly. The overly high proportion of secondary industries is not conducive to the development of the CCFE [59], especially after 2015. The improvement in regional technological innovation is conducive to CCFE development [60,61]. However, technological innovation shows a competitive effect that is especially obvious in the western region. Additionally, the high education resources in the eastern region are not conducive to the development of the CCFE, and the positive spatial spillover effect is significantly obvious in the eco-geographically adjacent province. In contrast, the high education resources in the central and western regions are conducive to the development of the CCFE. In addition, the proportion of fixed investments is not conducive to the development of the CCFE in the eastern region, western region, and northeastern region, showing a positive spillover effect in eco-geographically adjacent spaces.

Finally, according to the spatial Durbin model, we found the spatial spillover effects of different driving forces. The increase in the population urbanization rate promotes the development of the CCFE in the sample, significantly reducing ecology and natural resource pressures such as water for humans, similar to the findings of Kassouri (2021) [62]. The increase in the proportion of secondary industries is not conducive to the development of the CCFE, and the direct effect of the proportion of secondary industries is more obvious than the indirect effect. In contrast, the restricted effect of the proportion of secondary industries in the development of the CCFE is stronger in the external regions [63]. The improvement of technological innovation in the short term has a negative impact on the development of the CCFE, and the restricted effect on the local level is more obvious, promoting the development of the CCFE in eco-geographically adjacent provinces. In addition, the increase in the proportion of fixed investments is not conducive to the development of the CCFE in the overall sample and the eco-geographically adjacent provinces.

However, our study has several limitations. Firstly, the study period was from 2003 to 2020. For data limitation, we did not extend the data to 2021 or before 2003, and statistical bias may underestimate the development of the CCFE. Second, limiting data accessibility, green finance was not calculated by more micro-related indicators in the green finance field, such as provincial green investments and loans. This might have resulted in the underestimation of the green finance impact. Third, this article analyzed the macro driving force of the CCFE directly but did not emphasize the factors and mechanism of any aspect deeply. Therefore, we may have to empirically analyze the driving force of one or more micro aspects in the future.

## 5. Conclusions

This article analyzes the overall characteristics, spatiotemporal distribution patterns, and evolutionary characteristics of the spatial clustering and driving force of the CCFE, and the results can be concluded as follows:

(1) The CCFE in China is at the primary stage, with a fluctuating upward trend, showing spatial distribution characteristics, such as being higher in the eastern region and lower in other regions. Additionally, there are more regions with the types of primary coordination and basic un-coordination and fewer regions with the middle un-coordination and middle coordination types. Moreover, most regions dominated with the green finance lagged pattern, and fewer regions reached the finance-ecology synchronized pattern.
(2) The hot spots of the CCFE are located in the eastern region, such as the Pearl River, showing an “increase–decrease” development trend in the spatial space. Additionally, the cold spots of the CCFE are concentrated in the central and western regions, such as the Yellow River Basin, with a stably spatial space.
(3) There is a positive spatial spillover effect in the eco-geographically adjacent space in the development of the CCFE, with a significant cumulative transfer effect. The population urbanization and the number of granted patent applications have a significant positive impact on the CCFE, and the proportion of secondary industries has a significant negative impact. Spatially, the proportion of secondary industries and the number of granted patent applications have a negative impact on the development of CCFE in the eco-geographically adjacent space. Meanwhile, the influence and spatial effect of different factors on the development of the CCFE have an obvious heterogeneity in the different time and spatial ranges.

## 6. Policy Implications

Realizing the synchronized development of green finance and the ecological environment is an important direction of China’s green finance development and ecological environment protection in the “14th-Five-Year-Plan” period. Combined with the conclusions above, some policy implications are as follows:

(1) Continuously promote the coordinated development of green finance and the ecological environment. On one hand, in top-level rule design, it is necessary to take improvements in ecological environment quality as the fundamental goal and promote the quality development of green finance [64]. Green development should be closely integrated with the development targets of China in the 14th-Five-Year-Plan period. The production and service innovation of green finance should be combined with goals such as energy conservation, emission reduction, and clean production at each stage to place green finance into the micro construction circle of the ecological environment. On the other hand, it is necessary to accelerate green finance innovation to promote the high-quality construction of the ecological environment. In the national and provincial green finance reform and innovation demonstration zones, innovative experience as a model, production, service, and technology of green finance should be continuously explored to create a replicable and popularized model of coordinated development of green finance and the ecological environment, playing on positive cumulative transfer effects of CCFE.
(2) Classify and orderly complement the developmental shortcomings of green finance and the ecological environment. In one aspect, under the premise of continuously enhancing innovations such as green bonds and green securities, the eastern developed region should focus on the construction of the ecological environment, emphasize environmental issue governance such as soil erosion, soil salinization, water pollution, haze, and carbon emission, and supplement the infrastructure construction of rural environmental protection. Meanwhile, the eastern region should raise their regulatory standards and accelerate the transmission velocity of low-carbon industries. In other aspects, under the premise of reinforcing the construction of the ecological environment, the central and western regions should comprehensively use green financial tools such as green standards, environmental risk management [65], and fiscal discounts to improve the high-quality development of green finance. Especially in the key fields of current green technology, such as carbon capture and storage, they should strengthen the support of green finance.
(3) Strengthen the cooperation and the regional demonstration effect of coordinated development. China should take full advantage of the coordinated development of green finance and ecological environment development in different regions and deeply embed the coordination relationship between green finance and the ecological environment to create a healthy interactive development [66]. On one hand, it should continuously take advantage of the demonstration and leading effects of national green finance reform and innovation zones such as Guangzhou and Zhejiang, promote the green finance model, green credit resources, and green technological talent spillovers into the periphery region, and improve the high-quality development of green finance in adjacent regions. On the other hand, it should continuously take advantage of the demonstration and leading effects of national ecological environment zones such as Fujian, Jiangxi, Guizhou, and Hainan, replicate and promote the demonstration experience, such as water resource and environmental governance, rural living environment governance, ecological protection and restoration, ecological poverty alleviation, and ecological compensation, and improve the high-quality development of the ecological environment in adjacent regions.
(4) Fully release the element dividends, such as population, green industry, and technological innovation. Authorities in the eastern region, such as the Zhejiang and Guangdong authorities, should optimize the talent structure in green finance and the ecological environment and introduce high-level talent to the field of technological innovation and green corporation governance. The government in central and western regions should expand the talent scale in the coordinated development of green finance and the ecological environment and encourage and support the innovation and entrepreneurship of talent from eastern economically developed regions. Additionally, on the premise of adhering to the high-quality development of the real economy, the decision-makers should enhance the percentage of green industries, such as clean energy, green transportation, and green buildings, accelerate the green and low-carbon transformation of industrial structures [67], reduce emissions such as carbon emissions, and build a carrier for the coordinated development of green finance and the ecological environment. In addition, policymakers should strengthen innovation in technology, such as big data and cloud computing, promote the innovation of green service models and products, and improve the service efficiency of green finance. Moreover, it should enhance the basic research on applications such as the large-scale utilization of renewable energy, energy conservation, power batteries, and new power systems; accelerate technological innovation in electrical safety, high-efficiency photovoltaics, low-cost carbon dioxide capture, utilization and storage, and large-capacity energy storage; and improve the efficiency of different technology services for ecological environment construction.

## Figures and Tables

**Table 1:** Comprehensive appraisal index system and statistical descriptions.

Description	Unit	Mean	Std. Dev.	Min	Max
The market value proportion of environmental protection enterprises to total enterprises	%	1.479	2.485	0.000	22.427
The proportion of fixed asset investments in water conservancy, environment, and the public facilities management industry to total social fixed asset investment	%	10.269	5.234	3.484	45.650
The proportion of interest expenses in the output of six high-energy-consumption industries to the total industry	%	58.616	18.655	16.451	116.258
The proportion of completed investment in industrial pollution to GDP	%	0.147	0.131	0.001	0.992
Total industrial wastewater discharge	10,000 tons	71,570.25	59,639.56	3453.0	296,318.0
Total industrial waste air emission discharge	100 million standard m^3^	18,079.460	15,778.840	533.000	87,297.970
Total industrial solid waste production	10,000 tons	12,512.900	21,066.850	91.000	265,481.500
The number of environmental pollution incidents	times	22.248	41.412	0.000	406.000
The percentage of fiscal expenditure on environmental pollution to GDP	%	0.709	0.511	0.019	3.614
The percentage of environmental pollution governance investment to GDP	%	1.319	0.683	0.121	4.240
The harmless treatment rate of domestic waste	%	78.761	23.270	11.800	100.393
Total industrial wastewater treatment	10,000 tons	214,968.900	273,059.700	4213.491	2,125,782.000
The city sewage treatment rate	%	76.298	21.515	0.210	100.000
Treatment capacity of industrial waste gas treatment facilities	10,000 m^3^/h	118,491.00	301,661.10	449.361	3,190,759.0
Comprehensive utilization rate of industrial solid waste	%	66.100	20.374	15.903	99.600
The area of natural protection	10,000 hectares	356.988	560.576	9.038	2183.698
Public green area per capita	m^2^	11.033	3.457	3.100	21.795
Green space rate of built-up area	%	33.082	5.543	15.640	47.305
Forest coverage	%	30.768	17.791	2.940	66.971
Electricity consumption	100 million kwh	1556.901	1291.464	56.620	6940.000
Energy consumption	10,000 tons of standard coal	12,832.900	8441.495	684.000	42,441.410
Energy consumption per unit GDP	ton of standard coal/10,000 yuan	1.066	0.673	0.207	4.524
Total apparent CO_2_ emissions	mt	94.206	132.486	0.200	879.816
Carbon intensity	ton/10,000 Yuan	1.363	2.079	0.001	12.085

Note: (1) Data shown in Table 1 range from 2003 to 2020 in China; (2) the data source mentioned in Section 2.3.

**Table 2:** The CCFE type.

CCFE Level	Type	Efficacy	Pattern
0.8 < *iccdn_j_* ≤ 0.1	Quality coordination (TCC)	*S_iecolg_* < *S_igreen_*	Ecological environment lagged
		*S_iecolg_ ≈ S_igreen_*	Green finance and ecological environment synchronization
		*S_iecolg_ > S_igreen_*	Green finance lagged
0.6 < *iccdn_j_*≤ 0.8	High coordination (HCC)	*S_iecolg_* < *S_igreen_*	Ecological environment lagged
		*S_iecolg_ ≈ S_igreen_*	Green finance and ecological environment synchronization
		*S_iecolg_ > S_igreen_*	Green finance lagged
0.5 < *iccdn_j_* ≤ 0.6	Middle coordination (MCC)	*S_iecolg_* < *S_igreen_*	Ecological environment lagged
		*S_iecolg_ ≈ S_igreen_*	Green finance and ecological environment synchronization
		*S_iecolg_ > S_igreen_*	Green finance lagged
0.4 < *iccdn_j_* ≤ 0.5	Primary coordination (UCC)	*S_iecolg_* < *S_igreen_*	Ecological environment lagged
		*S_iecolg_ ≈ S_igreen_*	Green finance and ecological environment synchronization
		*S_iecolg_ > S_igreen_*	Green finance lagged
0.3 < *iccdn_j_* ≤ 0.4	Basic un-coordination (EUCC)	*S_iecolg_* < *S_igreen_*	Ecological environment lagged
		*S_iecolg_ ≈ S_igreen_*	Green finance and ecological environment synchronization
		*S_iecolg_ > S_igreen_*	Green finance lagged
0.2 < *iccdn_j_* ≤ 0.3	Middle un-coordination (MUCC)	*S_iecolg_* < *S_igreen_*	Ecological environment lagged
		*S_iecolg_ ≈ S_igreen_*	Green finance and ecological environment synchronization
		*S_iecolg_ > S_igreen_*	Green finance lagged
0 < *iccdn_j_* ≤ 0.2	Extreme un-coordination (DUCC)	*S_iecolg_* < *S_igreen_*	Ecological environment lagged
		*S_iecolg_ ≈ S_igreen_*	Green finance and ecological environment synchronization
		*S_iecolg_ > S_igreen_*	Green finance lagged

Note: *S_iecolg_* ≈ *S_igreen_* refers to |*S_iecolg_* − *S_igreen_*| ≤ 0.1.

**Table 3:** Urban resilience evaluation index system.

Variable	Mean	Std. Dev.	Min	Max	Obs.
lniccdn	−0.996	0.183	−1.644	−0.544	540
lnrurban	3.953	0.269	3.215	4.495	540
lnrsecd	3.789	0.225	2.760	4.119	540
lnrpatent	9.277	1.716	4.248	13.473	540
lnnedu	7.682	0.416	6.547	8.839	540
lnrfixed	4.158	0.432	2.359	5.250	540

Note: (1) ln refers to the logarithm of variables. (2) Std.Dev represents the standard deviation of the variable. (3) Obs. is the total number of samples.

**Table 4:** The pattern of CCFE in the years 2003, 2009, 2015, and 2020, respectively.

Pattern	2003	2009	2015	2020
Ecological environment lagged	Jiangsu, Guangdong, Zhejiang, Heilongjiang, Hubei, Chongqing, Guizhou, Tianjin, Shaanxi	Shaanxi, Guizhou, Jilin, Zhejiang, Guangdong, Hubei, Tianjin	Guizhou, Hubei, Tianjin, Chongqing	Heilongjiang, Anhui, Guangdong, Jiangxi, Zhejiang, Hubei, Hunan, Tianjin, Fujian, Shanghai, Shaanxi, Chongqing
Green finance and ecological environment synchronization	Jilin, Jiangxi, Sichuan, Ningxia, Henan	Chongqing, Henan	Shanghai, Shaanxi, Fujian, Zhejiang, Hunan, Guangdong, Sichuan, Heilongjiang	Sichuan, Beijing
Green finance lagged	Gansu, Liaoning, Shanxi, Shandong, Qinghai, Hebei, Guangxi, Anhui, Xinjiang, Inner Mongolia, Hainan, Hunan, Yunnan, Beijing, Shanghai, Fujian	Gansu, Qinghai, Shanxi, Inner Mongolia, Ningxia, Hebei, Shandong, Yunnan, Guangxi, Liaoning, Hainan, Xinjiang, Beijing, Hunan, Anhui, Jiangsu, Sichuan, Heilongjiang, Shanghai, Jiangxi, Fujian	Qinghai, Ningxia, Xinjiang, Shanxi, Yunnan, Shandong, Inner Mongolia, Hebei, Liaoning, Guangxi, Anhui, Hainan, Henan, Jiangsu, Jiangxi, Beijing, Jilin	Shanxi, Ningxia, Hainan, Qinghai, Yunnan, Shandong, Gansu, Xinjiang, Jiangsu, Hebei, Guizhou, Jilin, Liaoning, Henan, Inner Mongolia

**Table 5:** The estimation results of the benchmark model.

Variable	(1)	(2)	(3)	(4)	(5)
L.Wlniccdn	1.474 ***	1.585 ***	1.356 ***	1.018 **	0.859 *
	(3.78)	(4.09)	(3.33)	(2.30)	(1.94)
lnrurban	0.143 **	0.234 ***	0.183 **	0.228 **	0.274 ***
	(2.21)	(3.26)	(2.39)	(2.19)	(2.58)
lnrsecd		−0.166 ***	−0.172 ***	−0.159 ***	−0.162 ***
		(−3.29)	(−3.40)	(−3.05)	(−3.13)
lnrpatent			0.0217	0.0246	0.0281 *
			(1.35)	(1.50)	(1.72)
lnnedu				−0.0423	−0.0791
				(−0.84)	(−1.49)
lnrfixed					0.00875
					(0.48)
W×lnrurban	−1.582 ***	−1.198 *	−0.0603	−0.846	−0.120
	(−2.60)	(−1.77)	(−0.07)	(−0.81)	(−0.11)
W×lnrsecd		−1.203 *	−1.751 **	−2.108 ***	−2.185 ***
		(−1.80)	(−2.53)	(−2.92)	(−3.03)
W×lnrpatent			−0.384 **	−0.403 **	−0.373 **
			(−2.23)	(−2.29)	(−2.12)
W×lnnedu				0.932	1.059 *
				(1.52)	(1.66)
W×lnrfixed					−0.611 ***
					(−2.84)
Spatial rho	0.716 ***	0.694 ***	0.693 ***	0.753 ***	0.781 ***
	(2.68)	(2.61)	(2.62)	(2.79)	(2.88)
R2	0.004	0.053	0.022	0.067	0.072
N	510	510	510	510	510

Note: (1) *, **, *** represent significance at the 10%, 5%, 1% confidence levels. (2) The number in parentheses is the t-value. (3) L. refers to the first-order lag. (4) W is the spatial weight matrix. (5) N refers to the number of samples. (6) R2 refers to the coefficient of determination. (7) The chi2(5) value of the LR test in column (5) is 29.48, with a *p*-value equal to 0.0000, indicating that the SDM model chosen is more suitable than the others; in the other columns, this conclusion is still valid.

**Table 6:** The heterogeneity estimation results of influence factors.

Variable	(6)	(7)	(8)	(9)	(10)	(11)
	≤2014	>2014	East	Center	West	Northeast
L.lniccdn	0.566 ***	0.361 ***	0.436 ***	0.507 ***	0.464 ***	0.115
	(13.25)	(4.82)	(8.37)	(7.17)	(7.69)	(1.01)
lnrurban	0.00583	0.0472	0.424 *	−1.655 ***	−0.907 ***	1.869 *
	(0.04)	(0.17)	(1.77)	(−2.62)	(−2.67)	(1.70)
lnrsecd	−0.0715	−0.319 ***	0.0326	0.137	−0.191	0.351
	(−1.16)	(−3.18)	(0.28)	(0.82)	(−1.36)	(1.44)
lnrpatent	−0.00470	0.0194	−0.0124	0.0358	0.129 ***	−0.0288
	(−0.28)	(0.67)	(−0.49)	(0.80)	(2.76)	(−0.30)
lnnedu	−0.0178	0.0736	−0.312 ***	0.502 **	0.266 **	−0.400
	(−0.30)	(0.59)	(−2.84)	(2.39)	(2.09)	(−0.59)
lnrfixed	0.0346	−0.0833 ***	−0.155 ***	−0.0830	−0.137 *	−0.0758 **
	(1.05)	(−3.22)	(−2.82)	(−1.57)	(−1.66)	(−2.00)
W × lnrurban	−1.145 **	6.191 **	−0.325	1.516 *	1.044 *	−2.882 *
	(−2.15)	(2.41)	(−0.73)	(1.75)	(1.88)	(−1.69)
W × lnrsecd	−1.027 ***	−3.170 ***	−0.103	−0.412 *	−0.00163	−0.813 *
	(−4.46)	(−2.87)	(−0.66)	(−1.69)	(−0.01)	(−1.80)
W × lnrpatent	0.102	0.370	−0.0307	−0.0684	−0.183 **	0.0474
	(1.62)	(1.16)	(−0.90)	(−0.81)	(−2.18)	(0.35)
W × lnnedu	0.234 *	−0.754	0.361 **	−0.395	−0.327	0.429
	(1.81)	(−0.53)	(2.33)	(−1.52)	(−1.57)	(0.59)
W × lnrfixed	−0.107	−0.321	0.299 ***	0.196 *	0.332 **	0.225 **
	(−0.93)	(−1.09)	(2.67)	(1.91)	(2.07)	(2.08)
Spatial rho	0.535 ***	1.168 **	0.496 ***	0.397 ***	0.321 **	0.331 *
	(4.65)	(2.36)	(5.26)	(2.59)	(2.15)	(1.72)
R2	0.689	0.044	0.387	0.799	0.609	0.002
N	330	180	170	102	187	51

Note: (1) *, **, *** represents significance at the 10%, 5%, 1% confidence levels. (2) The number in parentheses is the t-value. (3) L., R2, W, N are similar to the above.

**Table 7:** The estimation result of the spatial spillover effect.

Variable	(12)	(13)	(14)	(15)	(16)	(17)
	SR_Direct	SR_Indirect	SR_Total	LR_Direct	LR_Indirect	LR_Total
lnrurban	0.268 ***	−0.188	0.080	0.266 **	−0.115	0.151
	(2.56)	(−0.28)	(0.11)	(2.09)	(−0.07)	(0.08)
lnrsecd	−0.167 ***	−1.183 ***	−1.350 ***	−0.233 ***	−2.664 *	−2.897 *
	(−3.29)	(−2.78)	(−3.06)	(−2.98)	(−1.6)	(−1.68)
lnrpatent	0.026 **	−0.218 **	−0.192 **	0.016	−0.428	−0.412
	(1.62)	(−2.1)	(−1.77)	(0.79)	(−1.39)	(−1.28)
lnnedu	−0.073	0.640	0.567	−0.043	1.261	1.218
	(−1.5)	(1.54)	(1.39)	(−0.71)	(0.98)	(0.92)
lnrfixed	0.006	−0.359 ***	−0.353 **	−0.012	−0.749	−0.761
	(0.34)	(−2.57)	(−2.46)	(−0.47)	(−1.53)	(−1.5)
lnrurban	0.268 ***	−0.188	0.080	0.266 **	−0.115	0.151
	(2.56)	(−0.28)	(0.11)	(2.09)	(−0.07)	(0.08)

Note: (1) *, **, *** represents significance at the 10%, 5%, 1% confidence levels. (2) The number in parentheses is the t-value. (3) SR and LR refer to the short-term and long-term periods, respectively.

## Data Availability

The data presented in this study are openly available from the China Statistical Yearbook, available at the website: http://www.stats.gov.cn/tjsj/ndsj, (accessed on 5 January 2022); the China Financial Statistics Yearbook, available at the website: https://data.cnki.net/yearbook/Single/N2022010064, (accessed on 3 January 2022); and the China Environmental Statistical Yearbook, available at the website: https://data.cnki.net/yearbook/Single/N2021070128, (accessed on 3 January 2022).
